# Detection of active transposable elements in *Arabidopsis thaliana* using Oxford Nanopore Sequencing technology

**DOI:** 10.1186/s12864-017-3753-z

**Published:** 2017-07-17

**Authors:** Emilie Debladis, Christel Llauro, Marie-Christine Carpentier, Marie Mirouze, Olivier Panaud

**Affiliations:** 1 Université de Perpignan Via Domitia, Laboratoire Génome et Développement des Plantes, 52, avenue Paul alduy, 66860 Perpignan cedex, France; 2 Centre National de la Recherche Scientifique, Laboratoire Génome et Développement des Plantes, 52, avenue Paul alduy, 66860 Perpignan cedex, France; 3 Institut de Recherche pour le Développement, UMR232 DIADE Diversité Adaptation et Développement des Plantes, Perpignan, France; 4 Institut Universitaire de France, Paris, France

**Keywords:** Transposable elements, *Arabidopsis*, Oxford Nanopore Technology, Sequencing

## Abstract

**Background:**

Transposables elements (TEs) contribute to both structural and functional dynamics of most eukaryotic genomes. Because of their propensity to densely populate plant and animal genomes, the precise estimation of the impact of transposition on genomic diversity has been considered as one of the main challenges of today’s genomics. The recent development of NGS (next generation sequencing) technologies has open new perspectives in population genomics by providing new methods for high throughput detection of Transposable Elements-associated Structural Variants (TEASV). However, these have relied on Illumina platform that generates short reads (up to 350 nucleotides). This limitation in size of sequence reads can cause high false discovery rate (FDR) and therefore limit the power of detection of TEASVs, especially in the case of large, complex genomes. The newest sequencing technologies, such as Oxford Nanopore Technologies (ONT) can generate kilobases-long reads thus representing a promising tool for TEASV detection in plant and animals.

**Results:**

We present the results of a pilot experiment for TEASV detection on the model plant species *Arabidopsis thaliana* using ONT sequencing and show that it can be used efficiently to detect TE movements. We generated a ~0.8X genome coverage of a met1-derived epigenetic recombinant inbred line (epiRIL) using a MinIon device with R7 chemistry. We were able to detect nine new copies of the LTR-retrotransposon Evadé (*EVD*). We also evidenced the activity of the DNA transposon CACTA, *CAC1*.

**Conclusions:**

Even at a low sequence coverage (0.8X), ONT sequencing allowed us to reliably detect several TE insertions in *Arabidopsis thaliana* genome. The long read length allowed a precise and un-ambiguous mapping of the structural variations caused by the activity of TEs. This suggests that the trade-off between read length and genome coverage for TEASV detection may be in favor of the former. Should the technology be further improved both in terms of lower error rate and operation costs, it could be efficiently used in diversity studies at population level.

**Electronic supplementary material:**

The online version of this article (doi:10.1186/s12864-017-3753-z) contains supplementary material, which is available to authorized users.

## Background

Transposable elements (TEs) are widespread in eukaryotic genomes. Because of their nature and ability to increase in copy number, TEs play a major role in the structure and evolution of the genome in most eukaryotic lineages [1, 2]. Transposable Elements-associated structural variants (TEASVs) are common in natural and domesticated populations and contribute to a large extent to their genomic diversity, while they also can influence gene expression [3, 4] and lead to phenotypic variations [5, 6]. Therefore, the estimation of the contribution of TEASVs to biological diversity in plant and animals has become one of the objectives of genomics today.

In the past decade, the emergence of Next Generation Sequencing (NGS) technologies based on Illumina platform has opened new perspectives for the development of new methods to conduct systematic genome-wide surveys of TEASVs. Several methods have been proposed for the detection of TEASVs using this technology [7, 8]. These are referred to as paired-end mapping (PEM), depth of coverage (DOC) and split-read mapping (SRM). PEM is based on the mapping of both ends of amplicons from Illumina libraries onto a reference genome. Structural variants are detected when both ends of an amplicon map at different locations in the genome (*i.e.* at a distance which is significantly larger than the average size of the inserts of the Illumina library). The PEM was developed in order to best exploit the short reads that are paired from the same amplicon. In plants, this method allowed the detection of active TEs in rice [9, 10] and *Arabidopsis thaliana* [11]. In the latter case, the authors identified a LTR-retrotransposon Évadé (*EVD*) which is active in an hypomethylated mutant plant. However, several authors pointed out that the mapping of short reads in complex genomes is not always reliable and can lead to high False Discovery Rate (FDR, see [12, 13]). This is mostly due to their presence at high copy number in genomes, thus challenging their detection in repeated regions. The DOC method consists in estimating the sequencing coverage of known TE families to eventually detect an increase in their copy number as a signature of transposition. It is therefore restricted to TEs that transpose via a copy and paste mechanism, such as LTR retrotransposons, LINEs or SINEs. The DOC method is very robust and can detect insertions in repeated regions, unlike the previous methods described above [14, 15]. However, its major limitation is that it does not provide any information regarding the position of new insertions.

The SRM method is based on the identification of sequencing reads that span the junction between a new TE insertion and its native insertion site. It is conceptually the most reliable methods for TEASV detection, but the short read length produced by the Illumina technology has so far impeded its reliable use for exhaustive TEASV detection. The recent improvement of the technology in this matter has however led some authors to successfully use the SRM method [16–18], but the short read length still does not allow any reliable detection of TE insertions in repeated sequences. The recent development of third generation technologies that allow the sequencing of a single molecule and can generate long reads could open new perspectives for TEASV detection through SRM : the two platforms available so far, *i.e.* Pacific Biosciences and Oxford Nanopore Technologies, indeed allow to generate kilobases-long reads that could improve the reliability of TEASVs detection by providing enough sequence information to unambiguously map new TE insertion sites.

The Nanopore technology was conceptualized three decades ago [19] but lead only five years ago to the commercial release of the smallest hand-held sequencers, called MinIon. This new Nanopore sequencer is based on DNA characteristics where the use of a positive electrical potential lead to an ionic current. As DNA molecules are negatively charged, they can translocate through a protein nanopore leading to changes in current that are used to determine the nucleotide base sequence [20,21]. So far, both Pacific Biosciences and Nanopore technologies have suffered from high sequencing error rate, which impedes the direct use of the sequencing data for genome assemblies. However, in the case of TEASV detection, the trade-off between sequencing length and high error rate may be in favor of the former since we anticipate that a read of several kilobases could be unambiguously mapped onto a reference genome even if sequencing is achieved with an error rate of 10–15%. In this report, we tested this hypothesis by sequencing the genome of a partially hypomethylated line of the plant species *A. thaliana* [11,22] in which the LTR retrotransposon *EVD* is transpositionally active. We indeed show that new insertions of *EVD* as well as of another TE family could easily and reliably be detected in this plant material.

## Results

### Characteristics of the raw dataset

A qPCR assay of the epiRIL12 line that we used in this study suggested the presence of newly transposed copies of the retrotransposon *EVD* (see Methods). This material was therefore perfectly suitable to tentatively detect these insertions using ONT sequencing platform. Three flow cells were used to sequence the genomic DNA of epiRIL12 line with two MinIon devices : one of the first available version and the other two of the MkI release type. The first MinIon run (FLO-MAP003) generated 49,061 sequence reads totalling 232 Mb. The ONT platform produces three types of reads : the template reads are produced from the forward strand sequencing. The complement reads are produced from the reverse strand sequencing. This distinction is made possible by the linking of the two strands of the molecule to be sequenced. The first strand to be sequenced is defined as the forward strand, then the second strand to be sequenced (from the same pore because it is physically linked to the first strand) is defined as the reverse strand. When both forward and complement reads are available for the same molecule, then the two sequences are merged into a single one, referred to as two direction reads or All 2D. For the first run, 98% of the molecules produced Template reads, while 40% produced both template and complement reads. Finally, 35% produced All 2D reads merged into a single consensus sequence. The last two runs (FLO-MAP103) produced 9,796 and 4,644 reads (66 and 38 Mb) respectively. 100% were template reads, with 6,437 and 3,008 complement reads, respectively. Of these, a total of 5,490 and 2,357 were converted into All 2D reads.

Because the reads obtained from the 3 MinIon sequencing runs corresponded to the same DNA sample of epiRIL12, all sequence data were concatenated into a final multifasta file containing 118,554 sequences. The read length varied between 6 and 691,915 nucleotides, although 72% of the reads had a length comprised between 500 and 15,000 nucleotides with a median size of 4.6 kb (Additional file 1: Figure S2).

All reads were then mapped onto the *Arabidopsis* reference genome TAIR10 (The *Arabidopsis* Information Resource, http://www.arabidopsis.org) using blastN programme (see material and methods) with allowed gaps. 47,765 reads (40.3%) produced a significant alignment and were kept for further analyses. These represent ~80 Mbp of sequences, *i.e.* ~0.8X genome coverage. In order to validate this estimation, we searched for the presence of 10 selected unique *Arabidopsis* genes in our dataset and found that 8 produced a significant alignment with at least one of the 47,765 reads (Additional file 2: Figure S3), which confirms the first estimation. The average sequence identity between the reads and the reference *Arabidopsis* genome sequence was 85.8%, which is close to the manufacturer's claim with the R7 chemistry (*i.e.* 88%).

### Detection of EVD insertions sites

**Fig. 1:**
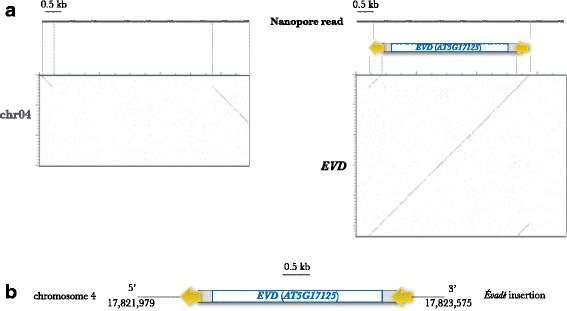
Characterization of EVD neo-insertion 4. **a** Dot-plots comparison of a 7 kb Nanopore read (horizontal) with the region of the insertion on chromosome 4 (left, vertical) and the sequence of EVD (right, vertical). Blue box : GAG-POL region, grey boxes : UTR and yellow arrows : long terminal repeats. **b** Schematic representation of EVD insertion in the chromosome 4. The orientation of the EVD LTRs are indicated by yellow arrows, the EVD insertion is in antisense

**Fig. 2:**
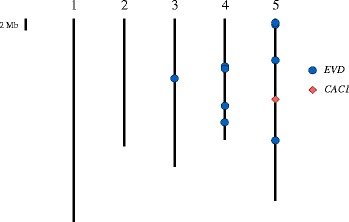
Map position of all neo-insertions detected in this study. The vertical bars represent the five chromosomes of Arabidopsis thaliana. The length of each chromosome is proportional to its physical size

The first step of our analysis was to search for all *EVD* insertions in the genome of the epiRIL12 line. For this, a simple similarity search of the *EVD* reference sequence used as a query against a database composed of 47,765 reads dataset, using the blastN programme was performed. Sixteen reads ranging from 4.2 to 8.8 kb showed similarity with *EVD*. Four of these reads entirely spanned the element (*i.e.* 100% of the reads corresponded to the element). Eleven reads showed partial similarity to *EVD*, with one part of the sequence corresponding to the element and the other to another locus in the *Arabidopsis* genome, which corresponded to nine distinct insertion sites. One among these nine insertion sites corresponded to the native copy of *EVD* present on the chromosome 5. Finally, one 7 kb read spanned the complete *EVD* sequence flanked by both insertion site sequences (Fig. 1). To our knowledge, this is the first example of the identification of a complete LTR-retrotransposon insertion in a single sequencing read. The map positions of the nine newly inserted *EVD* elements are represented in Fig. 2.

**Fig. 3:**
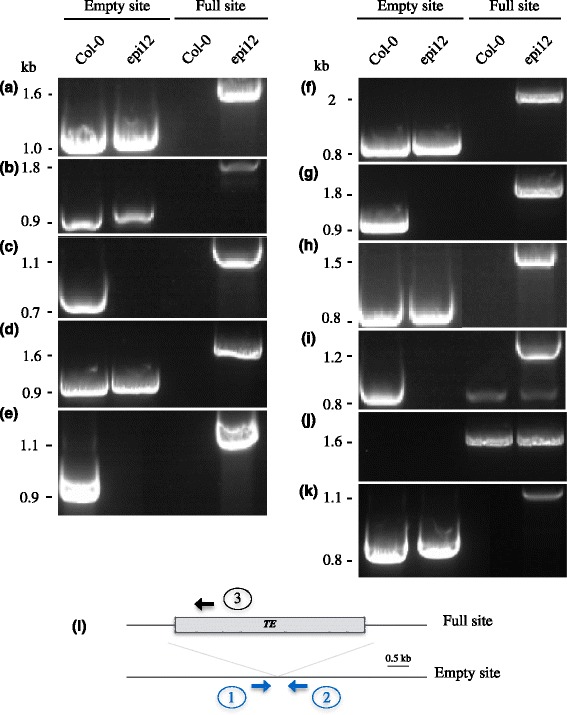
Validation of EVD insertions in epiRIL12 detected by MinION. PCR detection of EVD insertion polymorphism for neo-insertion 1 to 9 (**a** to **i**), the native copy of EVD on chromosome 5 as control (**j**), and the neoinsertion of CAC1 (**k**). **l** : schematic illustration of RBIP primer design. primers 1 and 2 are designed to detect empty sites and primers 1 and 3 (sense insertion) or 3 and 2 (antisense insertion) are designed to detect full sites

A wet-lab validation of *EVD* neo-insertion sites detected above was performed through PCR amplification and sequencing. All primers combinations confirmed the presence of the insertion at the expected location (Fig. 3 a to i). Five neoinsertions were found to be heterozygous (neo1, neo2,neo4, neo6 and neo8), while the remaining four are homozygous.

### Detection of transposition events ab initio

The long reads generated by the ONT platform should theoretically allow a reliable detection of TEASVs using the SRM method. Our method is based on blastN similarity search rather than on Burrows-Wheeler based algorithms usually used with Illumina data. BW-based algorithms are designed for the mapping of a high number of short reads onto a reference genome. Moreover, BW mapping is usually achieved at high stringency, given the short read length. The Nanopore reads are, on the contrary, very long and exhibit a lot of mismatchs and indels, due to the high error rate. We therefore found the BLAST algorithm more appropriate for the analyses. Thirty-six MinIon reads were identified as spanning a similarity breakpoint and homologous to one sequence of the *Arabidopsis* TE database. These putative transposition events were carefully checked manually using simple dotter alignments of the reads against both the TE sequence and the flanking sequence of the insertion, in order to eliminate false positives (see the discussion section). Only 10 reads were thus validated. Nine of these insertion sites corresponded to *EVD* transposition event, which is congruent with the results obtained using our first strategy (using the *EVD* sequence for similarity search). The other TEASV corresponded to the transposition of the CACTA transposon, *CAC1* (Fig. 2). These 10 insertions were validated through wet lab analyses (Fig. 3k)

## Conclusion/Discussion

In order to demonstrate the suitability of Nanopore technology for TEASV detection, we sequenced an epiRIL from *A. thaliana*. We used three runs of MinIon that in our hands produced from 38 to 232 Mbp, reaching a total of 336 Mb. This variation in yield was mostly attributed to differences in flow cell quality, according to the manufacturer's technical support. None of the flowcells provided subsequently indeed showed such discrepancies (personnal communication). After checking the quality of the sequences through homology searches against the *Arabidopsis* reference sequence, only 80 Mb, *i.en* 23% of the initial dataset could be used for further analyses. We do not provide any explanation for such large difference. Whether the non-*Arabidopsis* sequences originate from artefacts or from contaminants should be further tested. In addition, this result could not be attributed to the parameters used for the BLAST searches, as lowering the stringency of the searches did not modify the results. The latest flowcells and R9 chemistry developed by ONT appear to produce more reliable data. Further analyses are needed to confirm this.

The median of true *Arabidopsis* sequence reads was 4.6 kb. This represents a significant technological improvement compared to other NGS platforms and even compared to the Sanger-based techniques that could not generate reads larger than 700-800 nucleotides. In this regard, our results confirm that ONT could produce data of interest for the study of structural variants. We however first had to demonstrate that the transposition of the *EVD* retrotransposon could be detected through SRM. Using simple Blast homology searches of the reads again this TE, we were able to identify homology breakpoints and thus unambiguously map some of its insertions. Unfortunately, given the low amount of useful sequence data, we were not able to exhaustively count the number of new *EVD* insertions in this plant material. However, this first study leads us to conclude that despite a sequencing error rate of 13%, the reliable detection of *EVD* insertions was possible with only 0.8X genome coverage. Therefore, this pilot experiment constitutes a proof of concept that Nanopore technology is perfectly suitable for TEASV detection.

The real power of this new technology could only be demonstrated if it allows to identifiy exhaustively all transposition events in an individual without prior knowledge of active TE families. In the case of the *Arabidopsis* epiRIL that we tested, our ab initio search yielded the detection of a CACTA transposon insertion. This opens new perspective for the exhaustive characterization of active TEs in plants and animals. The identification of a 7 kb long read spanning a complete *EVD* element together with both sequences flanking its insertion indeed suggests that the Nanopore technology could greatly help identify active TEs in species for which high quality genomic resources (in particular genome assembly) are not available. This will however undoubtedly require the development of new Informatic tools specially designed for the analyses of such sequence data. Our analyses relied upon the use of Blast algorithm for the ab initio detection of TEASVs. This algorithm, although fast and powerful, may not be the best suited for such purpose. In particular, the split of the alignments in multiple HSPs at multiple loci (in case the sequence flanking the insertion of the TE contains repeats) is prone to FDR. We indeed had to validate our candidates both in silico (through manual examination of homologies) and through wet lab experiments. The use of algorithms that allow the sequence comparison of larger sequences (e.g. NucMer [22]) may considerably improve the efficiency of TEASV detection based on ONT data, although this has to be tested.

As we mentioned in the introduction, we anticipated that the trade-off between the error rate and the read length may be in favor of the latter. This is confirmed in our analysis. Although a direct comparison between ONT and Illumina platform on the same DNA source is needed to draw any conclusion on the superiority of one technology versus the other, the possibility to detect TEASVs with a 0.8X genome coverage is very promising, considering that, in the case of Illumina-based data, a genome coverage of at least 20X is necessary to reliably detect any TEASVs. We anticipate that a reliable identification of TEASVs could be achieved using low genome coverage, e.g. 2X, which may contribute to keep the detection cost to a reasonable level, as long as the technology is improved in terms of sequence reliability. Additional pilot experiments are also needed to confirm that ONT may be suited for the analyses of larger, more complex genomes. In any case, the development of single molecule sequencing technologies with long reads, such as ONT, opens new perspectives in many aspects of genomics. The reliable genome-wide characterization of structural variations, whether at individual level (e.g. somatic variations) or within populations, will help discover some new functional aspects of genome dynamics in plant and animals.

## Methods

### Plant material

*Arabidopsis thaliana* WT ecotype Columbia-0 and epiRIL12 plant materials previously described in [23], were used in this study. Plants were grown in soil under a 16 h/8 h (light/dark) cycle after 2 days at 4 °C for stratification.

### DNA preparation

Genomic DNA from epiRIL12 was extracted from seedlings ground to a fine powder in liquid N2. The powder was resuspended in 10 ml of CTAB2X extraction buffer (2% CTAB, 100 mM Tris-HCl pH 8, 20 mM EDTA pH 8, 1.4 M NaCl, 5% N-lauroylsarcosine di- sodium salt, 0.2% 2-mercaptoethanol) and incubated for 60 min at 65 °C. Then, an equal volume of chloroform was added and the emulsion was maintained during 10 min before centrifugation at 4,500 rpm for 10 min at room temperature. The nucleic acids were precipitated with isopropanol (0.7 v/v) at -80 °C for 15 min and centrifuged at 4 °C at 4,500 rpm for 45 min. Nucleic acids were further washed with 75% ethanol and centrifuged at 4 °C at 4,500 rpm for 10 min. Finally, the pellet was air dried and DNA was resuspended in 300 μl TE and was treated with 10 μg of RNase A (Qiagen, Hilden, Germany) for 30 min at 65 °C before further analysis.

### Estimation of EVD copy number

DNA was quantified using the Quant-iT dsDNA High sensitivity Assay (HS) Kit (Life Technologies) according to the manufacturer’s instructions. For qPCR analysis of *EVD* DNA copies, the Takyon No RoxSYBR MasterMix dTTP Blue Kit (Eurogentec, Liège, Belgium) was used and ACTIN2 was used to normalize DNA levels. DNA copy number was performed in a final volume of 10 μl employing a LightCycler® 480 (Roche, Basel, Switzerland). PCR were performed with primers summarize in Additional file 3: Table S1 with the following conditions: 95 °C for 5 min followed by 45 cycles of amplification composed of 10 s at 95 °C, 10 s at 60 °C and 10 s at 72 °C, then a final soak at 37 °C for 30 s was done. The *EVD* DNA copies were determined by the 2∆∆CT method and diagram represent relative quantity of amplification compared to the WT, which was taken as 2. Taking into account that the presence of extra-chromosomal forms of the element may induce a strong bias in the estimation of new *EVD* copies, we estimate that the line harbours at most 13 new insertions (Additional file 4: Figure S1).

### MinIon library preparation

The MinIon sequencing library was generated with the sequencing Kit (SQK-MAP-006) (Oxford Nanopore Technologies, Oxford, UK) according to the manufacturer’s instructions. Four microgram of DNA were fragmented as follows: DNA was loaded into a G-tube (Covaris, Brighton, UK) and spun at 6,000 rpm in an eppendorf 5424 for 1 min before inverting the tube to centrifuge again for 1 min. One microgram of fragmented DNA was end-repaired and dA-tailed using NEBNext Ultra II end-repair/dA-tailing module (Biolabs, New England, USA, cat. no. E7546S) as per the manufacturer’s instructions except for the thermal cycler program performed with 5 min at 20 °C, followed by 5 min at 65 °C and terminated with 5 min at 4 °C. The DNA was further purified with 1.0X vol. Agencourt AMPure XP beads (Beckman Coulter, High Wycombe, UK). After two washes with 70% of ethanol, beads were air dried and the DNA was elutated with 31 μl of Gibco distilled water. A ligation with biotinylated hairpin adapters was then performed by adding 50 μl Blunt/TA ligase master MIX (Biolabs, New England, US, cat. no. M0367S) to the A-tailed library. Library was enriched of molecules fixed with biotinylated hairpin thanks to the use of MyOneTM Streptavidin C1 Dynabeads (Thermofisher) prepared with bead binding buffer (BBB). Then, library was eluted off beads using 25 μl of elution buffer (ELB). The DNA concentration in the final library was quantified using a Quant-iT.

### Sequencing

Three Oxford Nanopore flow cells: 1 FLO-MAP003 and 2 FLO-MAP103 were used in this study. The number of available pores was first recorded to evaluate the flow cell’s quality with the MinKNOW™ software. The version v0.51.1.62 of MinKNOW™ software was used with the flow cell FLO-MAP003 and version v0.51.3.40 with the two other flow cells FLO-MAP103. Before starting the analyses, the Minknown application was first used to make the platform QC before starting the analyses and a map/NC-48-h sequencing run flow (Map 103) protocol was chosen and initiated. 500 μl of a priming mix (26.6 μl of Fuel mix (FMX), 500 μl of Running buffer (RNB), 473.4 μl of Nuclease-free water) were introduced two times in the MinIon flow cell to primed the sensory array. Thirty-six ng of the prepared library was diluted into a mix composed of 75 μl of RNB, 4 μl of FMX and Nuclease-free water was added to reach a final volume of 150 μl. The 150 μl priming mix was loaded into the sample loading port of the flow cell. The sequencing reaction was started. After 24 hours, 150 μl of a priming mix with 36 ng of the library was loaded again. One of the flow-cells FLO-MAP103 was loaded with a priming mix containing 108 ng of the prepared library. Base-calling was performed through data transference using the Metrichor™ agent (v2.39.3) and 2D base-calling workflow (v1.69). During the sequencing run, one additional freshly diluted aliquot of DNA library was loaded after 24 h of initial input.

### Characterisation of sequencing raw data

Raw reads were first extracted from the native HDF5 format MinIon reads using poretools [24] (Loman and Quinlan, 2014). Similarities between each read and *Arabidopsis* genome were estimated using a BlastN search using standard parameters, the read as query and the TAIR10 version of genome assembly as database. Reads producing alignment on TAIR10 assembly on at least 20% of their length were kept for further analyses. The estimation of the percentage of identity between the reads and the *Arabidopsis* genome was achieved by averaging that of all HSPs.

### Detection of EVD insertions

A BLAST multifasta database of all the MinIon reads was created with formatdb (version 2.2.26). Then the *EVD* sequence (ATCOPIA93, [11]) was aligned against the multifasta database with blastall (version 2.2.26) with the default parameters. For each blast hit, a dotter (version 4.1) with the Nanopore read against the *EVD* sequence was produced. In parallel, the same Nanopore read was aligned against the *Arabidopsis thaliana* genome, using ncbi BLAST with default parameters in order to identify the position of the flanking region. The start and end position of the alignement were kept and a sequence with plus or minus 1 kb was extracted from the *Arabidopsis thaliana* genome for each position. The extracted sequence and the *EVD* sequence were concatenated and a dotter with the 2D fasta MinIon read and the concatenated sequence was performed. Only the reads showing a clear similarity with the *EVD* and the flanking sequence, based on visual examination of the dotter output were kept for further characterization.

### ab initio detection

The ab initio detection method that we developed is conceptualy close to the Split Read Method developped for Illumina data, except that the long reads allow a systematic use of Blast algorithm. First, each read was aligned against TAIR10 genome assembly using BlastN using the -G 5 -E 2 -r 2 parameters in order to take into account the error rate generated by the technology. Those for which a maximum of 75% and a minimum of 400 bp contiguous sequences could be aligned were kept as putative representative of similarity breakpoints and then aligned against the *Arabidopsis* TE database (www.arabidopsis.org). Only the reads for which at least 200 bp of the remaining portion could be aligned on a well characterized TE were selected. After these filtering steps, each positive read was again compared to the putative region of the TE insertion (obtained by performing an extractseq of a 2 kb region spanning the insertion site) using the blast2seq software. Finally, only reads for which the sum of HSP length was higher than 300 bp and comprised between 33 and 75% of the total read length were kept. These candidates were finally checked manually using of dotter visualization (similarly to the procedure used for the identification of *EVD* insertions described above).

### Wet-lab validations

RBIP is a PCR-based marker strategy using a three-primer set derived from the flanking region and the LTR to the LTR-RT TE. Here, RBIP primer sets were designed to amplify the full sites between the internal TE sequence and its flanking sequence. Primers used to confirm the point of neo-insertion were designed with the Primer3 tool http://primer3.ut.ee/, 0.4.0 version [25] (Additional file 3: Figure S3). PCR was performed in a volume of 15 μl containing 3 μl of Gotaq® DNA polymerase Buffer (Promega, Madison, WI, USA), 8.05 μl water, 0.3 μl of 10 μM dNTPs , 0.5 μl of 10 μM of each primer, 0.75 U (0.15 μl of Gotaq® DNA polymerase and 2.5 μl of 1 ng/μl DNA. Amplification was conducted under the same parameters except for the elongation step depending of the expected amplicon size. Regions containing the entire *EVD* insertion was amplified with an elongation time of 5 min. All other parameters were identical to the classical PCR approach. The amplification was performed with the program described as follows: Initial hot start (3 min, 94 °C) and then 30 cycles with denaturation (30 s, 94 °C), annealing (30 s, 62 °C), elongation (1-5 min, 72 °C), with a final soak (4 min, 20 °C). The amplified product was electrophoresed at 100 V on a 1% agarose gel (Euromedex, Souffelweyersheim, France) in a 0.5X TAE buffer (20 mM Tris, 10 mM acetate, 1 mM EDTA pH 8.3). The 1% agarose gel solution was prepared with Gel redTM 10,000X stock reagent (Interchim, Montluçon, France) diluted at 1:10,000. PCR amplicons were purified with the Geneclean® purification kit (MP Biomedicals, Santa Ana, CA, USA) according to the manufacturer’s instructions. Sanger sequencing was performed with the same primers used to obtain these PCR amplicons on the ABI3130X1 Genetic Analyser sequencer (Applied Biosystems, Thermo Scientific, Waltham, MA, USA) at the Sequencing platform of the LGDP, Perpignan, France. Analysis were then performed using the Ridom TraceEdit software (Ridom Bioinformatics, Würzburg, Germany).

## Additional files

Additional file 1 **Figure S2**. Box-plot of MinION read length distribution. (PDF 24 kb)

Additional file 2 **S3 text file**. Genomic sequences of 10 unique loci used to estimate the genome coverage of sequence data. Sequences are given in fasta format. (TXT 40 kb)

Additional file 3 **Table S1**. Primers used for quantitative PCR and for real-time PCR. (XLS 64 kb)

Additional file 4 **Figure S1**. Accumulation of EVD DNA. DNA accumulation of EVD for wild type (WT) and epiRIL12 (epi12) measured by qPCR (mean ± s.e.m., *n* = 3 technical repetitions). (PDF 16 kb)

## Abbreviations

DOC: Depth Of Coverage.
EpiRIL: Epigenetic Recombinant Imbred Line.
EVD: Evadé.
FDR: False discovery rate.
LTR: Long Terminal Repeat.
MULE: Mu-Like Element.
NGS: Next generation sequencing.
ONT: Oxford Nanopore Technology.
PCR: Polymerase Chain Reaction.
PEM: Paired End Mapping.
SRM: Split Read Mapping.
TE: Transposable Element.
TEASV: Transposable Elements-associated Structural Variants.
