# Supplementary figures and images for: Detection of active transposable elements in Arabidopsis thaliana using Oxford Nanopore Sequencing technology
Source: BMC Genomics. 2017 Jul 17;18:537. doi: 10.1186/s12864-017-3753-z (PMC5513335; doi:10.1186/s12864-017-3753-z)

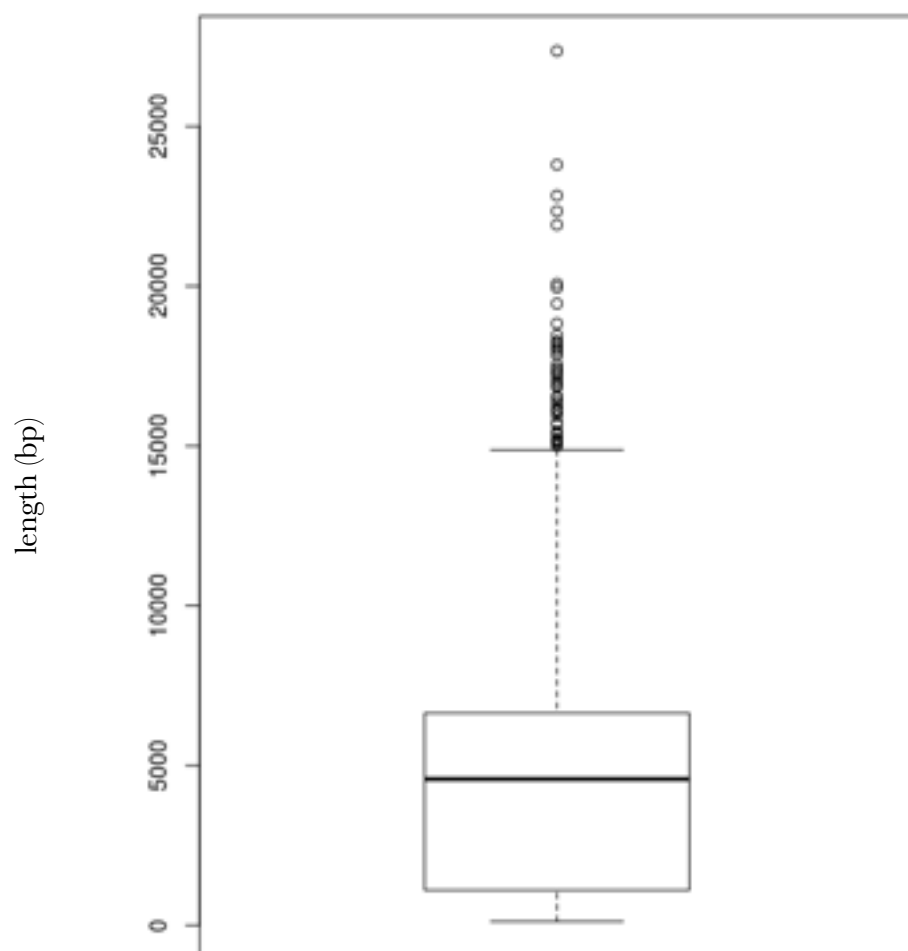

Figure S2.

Supplement: Supplementary file 1 — Figure S2. Box-plot of MinION read length distribution. (PDF 24 kb) [file 12864_2017_3753_MOESM1_ESM.pdf]

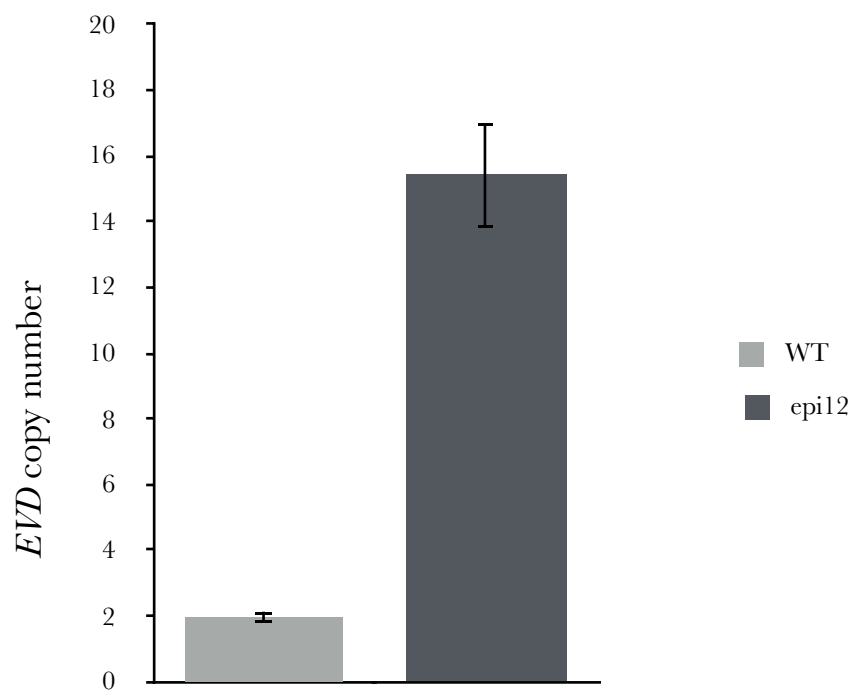

Figure S1.

Supplement: Supplementary file 4 — Figure S1. Accumulation of EVD DNA. DNA accumulation of EVD for wild type (WT) and epiRIL12 (epi12) measured by qPCR (mean ± s.e.m., n = 3 technical repetitions). (PDF 16 kb) [file 12864_2017_3753_MOESM4_ESM.pdf]
